# Limited hybridization between introduced and Critically Endangered indigenous tilapia fishes in northern Tanzania

**DOI:** 10.1007/s10750-018-3572-5

**Published:** 2018-04-18

**Authors:** Stephanie J. Bradbeer, Jack Harrington, Henry Watson, Abrahim Warraich, Asilatu Shechonge, Alan Smith, Rashid Tamatamah, Benjamin P. Ngatunga, George F. Turner, Martin J. Genner

**Affiliations:** 1 School of Biological Sciences, University of Bristol, Life Sciences Building, 24 Tyndall Avenue, Bristol, BS8 1TQ UK; 2 School of Biology, University of Leeds, Miall Building, Leeds, LS2 9JT UK; 3 Tanzania Fisheries Research Institute (TAFIRI), P.O. Box 9750, Dar es Salaam, Tanzania; 4 Department of Aquatic Sciences and Fisheries, University of Dar es Salaam, P.O. Box 35064, Dar es Salaam, Tanzania; 5 Evolutionary and Environmental Genomics Group, School of Environmental Sciences, University of Hull, Hull, HU5 7RX UK; 6 School of Biological Sciences, Bangor University, Bangor, Gwynedd, LL57 2UW UK

**Keywords:** Cichlid fish, Introgression, Conservation, Freshwater fish, Invasion biology

## Abstract

Hybridization between introduced and indigenous species can lead to loss of unique genetic resources and precipitate extinction. In Tanzania, the Nile tilapia (*Oreochromis niloticus*) and blue-spotted tilapia (*Oreochromis leucostictus*) have been widely introduced to non-native habitats for aquaculture and development of capture fisheries. Here, we aimed to quantify interspecific hybridization between these introduced species and the indigenous species *Oreochromis esculentus*, *Oreochromis jipe* and *Oreochromis korogwe*. In the Pangani basin, several hybrids were observed (*O. niloticus* × *O. jipe*, *O. leucostictus* × O. *jipe*, *O. niloticus* × *O. korogwe*), although hybrids were relatively uncommon within samples relative to purebreds. Hybrids between the native *O. jipe* × *O. korogwe* were also observed. In the Lake Victoria basin, no evidence of hybrids was found. Analysis of body shape using geometric morphometrics suggested that although purebreds could be discriminated from one another, hybrids could not be readily identified on body and head shape alone. These results provide the first evidence of hybridization between the introduced species and the Critically Endangered *O. jipe* in Tanzania. Given uncertainty regarding benefits of introduced species over large-bodied indigenous species in aquaculture and capture fisheries, we suggest that future introductions of hybridization-prone species should be carefully evaluated.

**Electronic supplementary material:**

The online version of this article (10.1007/s10750-018-3572-5) contains supplementary material, which is available to authorized users.

## Introduction

Introduced species are recognized as a major driver of biodiversity loss in aquatic environments (Clavero & García-Berthou, 2005), and many aquatic introduced species have been associated with substantial economic and ecological impacts (Lowe et al., 2000; Pimentel et al., 2005; Lowell et al., 2006). The spread of these species is predicted to continue as natural biogeographic barriers are overcome, either accidentally through unintended transport, or through deliberate introductions (Levine & D’Antonio, 2003; Taylor & Irwin 2004; Hulme et al., 2008; Westphal et al., 2008). Globally, there are few regions that have not been invaded by at least one introduced aquatic species (Molner et al., 2008; Leprieur et al., 2008), with global hotspots being highly correlated with human activity and trade (Drake & Lodge, 2003; Perrings et al., 2005; Meyerson & Mooney, 2007). Freshwater environments are considered especially vulnerable to invasion (Sala et al., 2000; Cox & Lima, 2006). A key concern is the inability to reverse invasions and the subsequent impacts, and only a few species have ever been successfully eradicated from an aquatic environment after establishment (Hill & Cichra, 2005; Williams & Grosholz, 2008; Leprieur et al., 2009; Hill & Sowards, 2015).

The range of ecological impacts associated with introduced species is extensive, including predation, competition and habitat alteration (Canonico et al., 2005). There is also growing concern surrounding the threat of the loss of indigenous unique genetic diversity through hybridization between native and introduced species, potentially even to the extent of species extinction (Levin et al., 1996; Rhymer & Simberloff, 1996). Hybridization is of particular concern in cases where one of the species are considered to be a threatened species. For example, in North America, vulnerable endemic Pecos pupfish *Cyprinodon pecosensis* (Echelle & Echelle 1978) have hybridized with the invasive sheepshead minnow *Cyprinodon variegatus* (Lacepède 1803) with potentially no pure populations remaining due to the apparent vigour of hybrid individuals (Rosenfield et al., 2004).

Hybridization has been invoked as a potential driver of biodiversity loss in the tilapiine cichlid fish of the genus *Oreochromis*. Several species have been introduced to non-native habitats in Africa with a view to developing and improving capture fisheries and aquaculture. Among the most widely distributed species is the Nile tilapia (*Oreochromis niloticus* L. 1758), native to the Nile basin and West Africa (Trewavas, 1983), but introduced into at least 15 African countries outside its native range (FAO, 2012). *Oreochromis* species are known for interspecific hybridization (Scribner et al., 2001), and hybrids are commonplace in aquaculture where they are selected for desirable characteristics such as salinity tolerance (Agresti et al., 2000; Kamal & Mair, 2005) and pigmentation (McAndrew et al., 1988; Romana-Eugia et al., 2004). In the natural environment, introduced *O. niloticus* has been documented as hybridizing with several species including *Oreochromis mossambicus* (Peters 1852) in South Africa (D’Amato et al., 2007), *Oreochromis aureus* (Steindachner 1864) in Egypt and West Africa (Rognon & Guymard, 2003; Bakhoum et al., 2009), *Oreochromis andersonii* (Castelnau 1861) and *Oreochromis macrochir* (Boulenger 1912) in Zambia (Deines et al., 2014), and *Oreochromis esculentus* (Graham 1928) and *Oreochromis leucostictus* (Trewavas 1933) in Kenya (Nyingi & Agnèse 2007; Angienda et al., 2011; Ndiwa et al., 2014). However, the underlying factors mediating levels of hybridization between *Oreochromis* are poorly understood.

In southern and eastern Africa, *O. niloticus* is now present in multiple major drainage systems where it was historically absent, including the Pangani, Rufiji, Ruvuma, Limpopo, Zambezi and Lake Victoria basins (Genner et al., 2013; Zengeya et al., 2013; Deines et al., 2014; Shechonge et al. unpublished data). This spread of *O. niloticus* has been accompanied by blue-spotted tilapia (*O. leucostictus*), native to Lakes Edward, George and Albert in the Nile system (Trewavas 1983; Shechonge et al. unpublished data). The consequences of introducing these species for native fauna in their new range are largely unknown (Deines et al., 2016). Concerns exist for both the conservation of native endangered populations (Gregg et al., 1998; Moralee et al., 2000; Nyingi & Agnèse, 2007; Angienda et al., 2011) as well as the preservation of wild genetic resources for aquaculture purposes (Lind et al., 2012).

In this study, we focus on the extent of hybridization between native and introduced *Oreochromis* in the Lake Victoria and Pangani basins of northern Tanzania. Both basins are characterized by endemic species that are threatened by negative interactions, including hybridization with introduced *O. niloticus* and *O. leucostictus*. Specifically, in the Lake Victoria basin, the Critically Endangered endemic *O. esculentus* persists only in satellite water bodies of Lake Victoria, although it has been translocated to multiple locations in surrounding countries (Trewavas, 1983; Shechonge et al. unpublished data). In the Pangani system, the Critically Endangered endemic *Oreochromis jipe* (Lowe 1955) is distributed from Lake Jipe to the Pangani Falls dam where it co-occurs with the lowland native species *Oreochromis korogwe* (Lowe 1955) (Trewavas, 1983; Shechonge et al. unpublished data). Given an apparent threat to Critically Endangered species, here we assess the extent of hybridization with introduced *Oreochromis* species using genetic (microsatellite) markers. We also asked if genetically identified hybrids could be identified using geometric morphometric data that capture variation in external head and body shape, potentially informing future field survey work on hybrid abundance.

## Methods

### Sampling

**Fig. 1:**
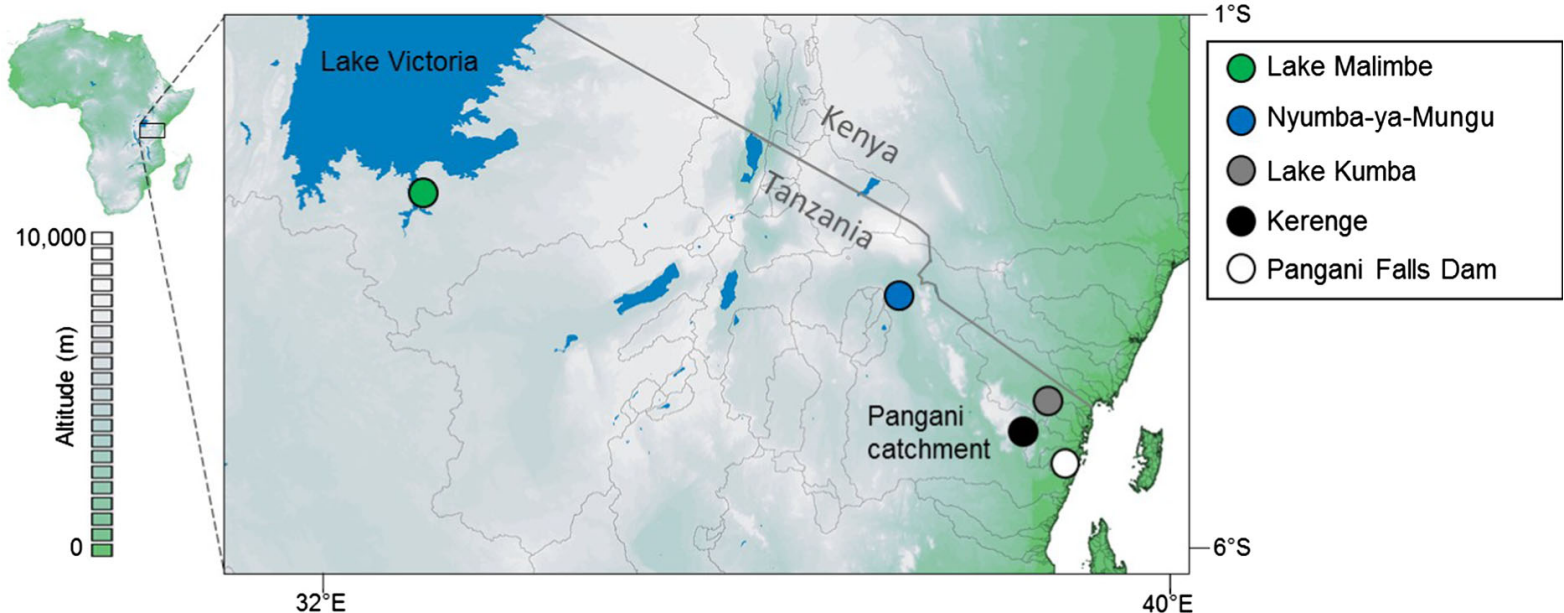
Locations of sampling sites in the Lake Victoria and Pangani basins

We collected our focal *Oreochromis* samples from one site in the Lake Victoria catchment and four sites in the Pangani river drainage (Fig. 1; Table 1). Sampling took place in 2015 and 2016 using seine nets, gill nets and purchasing from artisanal fisherman. Samples of additional reference species were obtained from five other sites in Tanzania (Table 1), where the species were found alone, or co-existing with species without any morphological or genetic evidence of hybridization. Fish captured using netting were immediately euthanized on landing by an overdose of clove oil anaesthetic. Specimens were pinned out and imaged in the field prior to preservation in absolute ethanol. Whole fish were placed in 70% ethanol for long-term storage. Genetic samples (fin clips) were taken and preserved in absolute ethanol.

**Table 1:** Sampling locations and sample sizes

Category	Date sampled	Site name	Latitude (°S)	Longitude (°E)	*N* individuals
Focal	12/08/2015	Kerenge	5.032	38.548	40
	12/08/2015	Lake Kumba	4.806	38.621	84
	14/08/2015	Nyumba ya Mungu	3.612	37.459	37
	19/08/2015	Pangani Falls dam	5.347	38.645	43
	04/08/2016	Lake Malimbe	2.627	32.899	56
Reference	13/08/2015	Lake Kalimawe (*O. jipe*)	4.422	38.089	13
	02/08/2016	Mwamapuli Dam (*O. niloticus*)	4.289	33.789	8
	18/08/2015	Mlingano dam (*O. korogwe*)	5.122	38.857	40
	02/09/2012	Lake Rukwa (*O. esculentus*)	8.397	32.901	7
	04/08/2016	Lake Victoria (*O. leucostictus*)	2.584	32.899	5

### Microsatellite genotyping

DNA was extracted from fin tissues using the Promega Wizard DNA extraction kit. Samples were screened at 17 microsatellite loci (Supporting Information Table 1). PCR was performed in a volume of 10 μl PCR solution consisting of 1 μl DNA (~ 5 ng), 5 μl Mastermix and 4 μl primer mix (10 mM). PCR amplifications were conducted on BioRad MyCycler thermal cycler, with conditions consisting of one denaturation step of 15 min at 95°C, followed by 35 cycles of 30 s at 94°C, 90 s at 57°C and 1 min at 72°C, followed by a final extension step of 30 min at 60°C. PCR products were sized on an ABI 3500 automated sequencer against a LIZ 500 size standard using GeneMapper 4.1 (Applied Biosystems).

### Microsatellite data analysis

All individuals were amplified at a minimum of 11 of the 17 microsatellite markers (Supporting Information Tables 2 & 3). Genetic diversity estimates, and tests of deviation from Hardy–Weinberg Equilibrium, were calculated in Arlequin 3.5 (Excoffier & Lischer, 2010). To estimate the genetic composition of individuals within sites, we used a two-step process. First we made an initial assignment of focal and reference individuals to species group using find clusters in the R package adegenet (Jombart & Ahmed, 2011), selecting the maximum number of Principal Components possible, and a K value reflecting the number of species present, based on their phenotypes. We then used this initial assignment to groups as prior (LOCPRIOR) in Structure 2.3.4 (Pritchard et al., 2000), selecting the admixture model, and 10 runs, each with a burn-in of 100,000 steps and 100,000 recorded iterations. Next, Clumpak (Kopelman et al., 2015) was used to summarize the Structure output. Individuals with an assignment probability lower than 0.9 to any one of the focal species were considered of hybrid origin.

### Geometric morphometrics

**Fig. 2:**
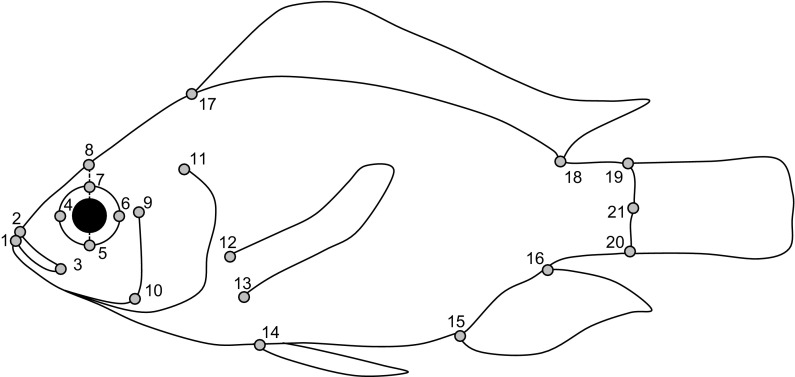
Landmarks used in geometric morphometric analyses

The left-hand side of the specimens was photographed with a scale. Images were loaded into tpsDIG v.2.22 (Rohlf, 2005) and 21 landmarks were digitized (Fig. 2). Landmarks were chosen based on landmarks commonly used in morphometric studies (Genner et al., 2007). Shape was quantified using MorphoJ 1.06 (Klingenberg, 2011). In each analysis, a Procrustes superimposition was applied to landmarked data. To visualize shape differences among purebred individuals at the sites, we used a Canonical Variates Analysis in MorphoJ 1.06. To determine repeatability of landmarking, we landmarked a set of 24 randomly selected individuals a second time. Following Procrustes alignment in MorphoJ 1.06, coordinates were subjected to Procrustes Anova the R package Geomorph (Adams et al., 2017) that revealed 54.85% of the total variance to be among individuals, 0.05% of the total variance between sets (original vs. repeat), and 45.10% representing residual variance.

To determine if hybrid individuals possessed intermediate morphology between parent species, we used a discriminant function analysis on procrustes scores in SPSS v.23.0 (IBM), using stepwise variable removal process that retained only the most informative variables. Genotypically “purebred” individuals were assigned to species groups, while putatively hybrid individuals were left unclassified. Individuals from Lake Kumba and Kerenge were pooled for morphological analyses due to their close geographic proximity and overlapping species composition. Individuals from the other three sites were analysed separately.

## Results

### Microsatellite results

**Fig. 3:**
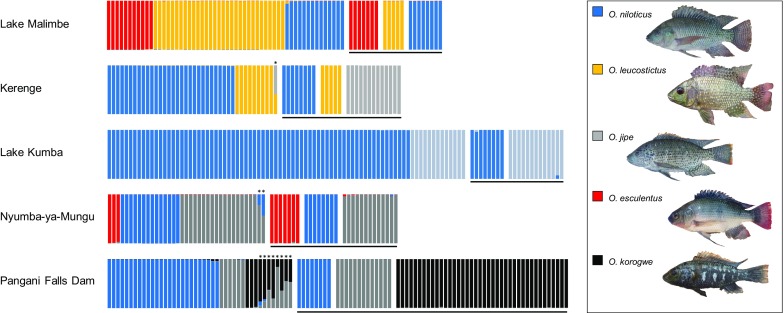
Posterior probabilities of assignment by structure to species groups of individuals collected at the five study sites, relative to the putatively purebred reference individuals. Each individual is represented by on vertical bar, with colours representing assignment probabilities to the species group. Reference individuals are underlined in black. Asterisk indicates putatively hybrid individuals

At each of the five sites, the complete samples showed significantly higher heterozygosity relative to expectations from Hardy–Weinberg equilibrium at most loci (Supporting Information Table 2). Analyses of these data supported the presence of *O. niloticus* at all five sites (Fig. 3), Lake Malimbe (*n* = 14), Kerenge (*n* = 30), Lake Kumba (*n* = 71), Nyumba-ya-Mungu (*n* = 14) and Pangani Falls Dam (*n* = 26). *O. leucostictus* was resolved as present at two sites, Lake Malimbe (*n* = 31) and Kerenge (*n* = 9). *O. esculentus* was present at two sites, Lake Malimbe (*n* = 11) and Nyumba-ya-Mungu (*n* = 3). *O. jipe* was present at three sites, Lake Kumba (*n* = 13), Nyumba-ya-Mungu (*n* = 18) and the Pangani Falls Dam (*n* = 6). *O. korogwe* was only found at the Pangani Falls Dam (*n* = 3). We found no evidence of hybrid individuals within Lake Malimbe or Lake Kumba. Evidence supporting the presence of hybrids was found at three sites (Fig. 3). One hybrid individual was identified at Kerenge sample (*O. leucostictus* × *O. jipe*). Two *O. niloticus* × *O. jipe* hybrids were in the Nyumba-ya-Mungu sample, and eight hybrid individuals were found in the sample from Pangani Falls (one *O. korogwe* × *O. niloticus*, and seven *O. korogwe* × *O. jipe*).

### Morphological characterization of purebred and hybrid individuals

**Fig. 4:**
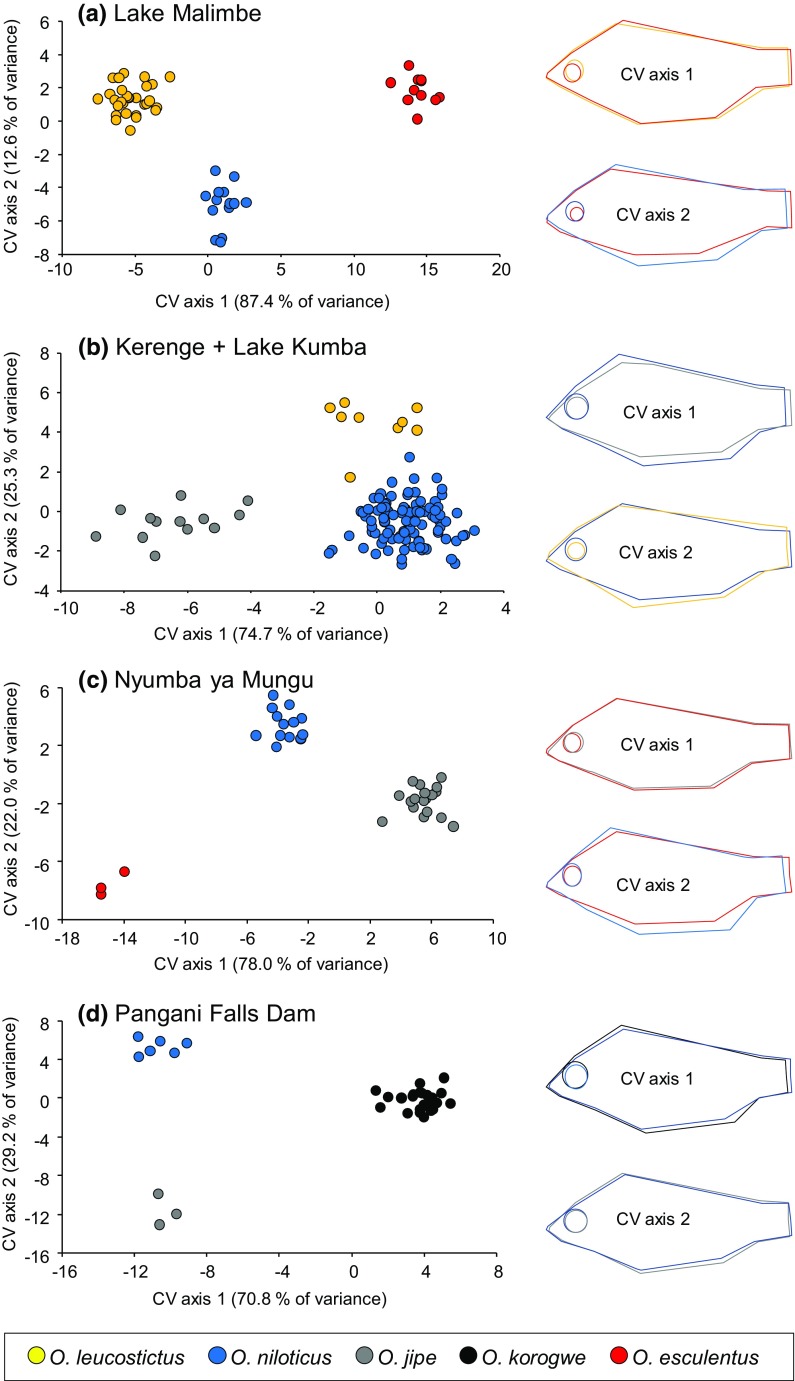
Canonical variate analysis of geometric morphometric shape variation among individuals identified as purebred. Individuals from Kerenge and Lake Kumba are grouped. Outline diagrams demonstrate the variation along CV axes, with line colours representing the species at each of the extremes of the axis

**Fig. 5:**
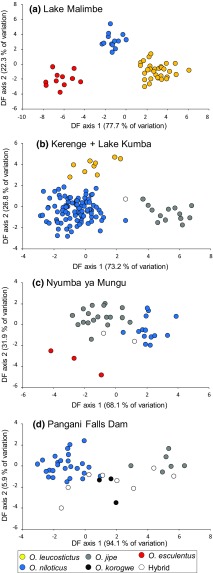
Discriminant function analysis of geometric morphometric shape variation among all individuals. Genetic purebreds were assigned to species, while hybrids were not preassigned. Individuals from Kerenge and Lake Kumba are grouped

Geometric morphometrics demonstrated significant differences in geometric morphometric space among purebred individuals (Table 2). Both CVA and discriminant analysis were consistent with sympatric populations being separable on shape variables (Figs. 4, 5), with evident differences primarily in body depth and eye size among species. In discriminant analyses, individuals identified as genetic hybrids were typically, but not exclusively, within the phenotypic space of parental purebred individuals (Fig. 5).

**Table 2:** Tests of multivariate shape differences among purebred populations using the variables retained in the reduced stepwise model used in the discriminant analysis

Site	Function	Wilks’ *λ*	*χ* ^2^	df	*P*
Kerenge + Lake Kumba	1 through 2	0.100	265.09	22	< 0.001
	2	0.443	93.64	10	< 0.001
Nyumba-ya-Mungu	1 through 2	0.092	71.57	10	< 0.001
	2	0.394	27.93	4	< 0.001
Pangani Falls dam	1 through 2	0.099	69.38	10	< 0.001
	2	0.717	9.97	4	0.041
Lake Malimbe	1 through 2	0.014	212.88	16	< 0.001
	2	0.201	79.39	7	< 0.001

## Discussion

We found no evidence of hybridization between *O. esculentus*, *O. niloticus* and *O. leucostictus* at any sites where pairs of these species co-occurred, including Lake Malimbe in the Lake Victoria catchment. Within the Pangani system, we found evidence for the presence of hybrid individuals of the Critically Endangered *O. jipe* with introduced species at two locations. At Kerenge one individual *O. leucostictus* × *O. jipe* was discovered, while at Nyumba-ya-Mungu two *O. niloticus*  × *O. jipe* were found. Notably, at all these sites hybrid individuals between introduced and native species were uncommon relative to purebred individuals. The relatively low frequency of hybridization in some of our sites is consistent with evidence from other *Oreochromis* systems, such as introduced *O. leucostictus* and native *O. nilo*ticus in Kenya (Nyingi & Agnèse, 2007; Ndiwa et al., 2014). These patterns contrast with observations of extensive hybridization between introduced *O. niloticus* and native *O. mossambicus* in South Africa (D’Amato et al., 2007), and between introduced *O. niloticus* and native *Oreochromis* in Zambia (*O. macrochir* and *O. andersonii*; Deines et al., 2014).

The relative rarity of hybrids between introduced and indigenous species can perhaps be explained by strong prezygotic isolating mechanisms. Sexual selection acting on male traits such as breeding colour, courtship displays and spawning “bower” phenotypes have all been suggested to promote reproductive isolation in mouthbrooding cichlids (Seehausen et al., 1997, 2008). Additionally, there is a possibility that populations may mate assortatively due to separate breeding periods or different breeding habitat preference, but at present no information on the habitat choice of these species is available. Postzygotic mechanisms may also have contributed to an apparent absence of hybrid individuals in our samples, if hybrid individuals suffer from low viability or fertility relative to purebred individuals. The extent of viability and fertility among the *Oreochromis* in our study is uncertain, although we note that hybrids of several *Oreochromis* species have been generated within aquaculture producing viable and fertile offspring (Bartley et al., 2001). In principle, the extent of inviability will be dependent on the extent of genomic incompatibility linked to the timescale since divergence (Bolnick & Near, 2005; Stelkens et al., 2009). However, the fitness of hybrid fish will also be dependent on the specific phenotypes of hybrids relative to the parental lines within the local selective regime. For example, phenotypically intermediate hybrids of cyprinids (Nilsson et al., 2017) and cichlids (Maan et al., 2017) have been demonstrated to have reduced survival relative to parental forms, while hybrids of two centrarchid species have an inferior feeding performance relative to their parental species (McGee et al., 2015).

The evidence of hybridization between the sympatric native species *O. korogwe* and *O. jipe* was notable in the Pangani Falls Dam, constructed in 1994. Surveys have reported these species that are otherwise allopatric in their distributions, with *O. korogwe* being distributed in low altitude coastal stretches of the Pangani and Zigi rivers, while *O. jipe* is primarily a higher altitude inland species (Trewavas, 1983; Shechonge et al., unpublished data). Further work is needed to map the distributions of both species within the lower reaches of the Pangani river system. It is possible that there is a natural hybrid zone, but it is possible that hybridization has been promoted by the habitat modification either linked to the dam construction, or the presence of *O. niloticus*. Dam construction has resulted in hybridization in other freshwater fishes (Hasselman et al., 2014), while introduced species have been suggested to affect the natural reproductive behaviour of native fish species (Doupe et al., 2009), including driving the loss of unique genetic diversity though hybridization (Velema et al., 2012).

### Timescale of spread of non-native species

An important factor determining the extent of hybridization and negative ecological effects on native fauna is the timescale of invasion. If hybridization is dependent on the density of the invader, then evidence of hybridization may be absent or rare until the invader becomes established and passes a threshold density, resulting in a lag time between the appearance of the non-native species and production of hybrids (Crooks & Soulé, 1999). *Oreochromis niloticus* and *O. leucostictus* are likely to have been first introduced into the Pangani system within the last 40 years. *Oreochromis niloticus* was notably absent from extensive surveys of Nyumba-ya-Mungu in 1974 (Bailey et al., 1978). At that time, *O. esculentus* was already established and abundant in the dam and forming an important part of the fishery. Given that *O. esculentus* had been reared in ponds in the lower Pangani near Korogwe as early as 1950 (Lowe-McConnell, 2006), it is possible that it arrived in the upper Pangani region prior to construction of the Nyumba-ya-Mungu dam in 1967–1969, and expanded in population size due to favourable conditions. Introductions of *O. leucostictus* and *O. niloticus* into Lake Victoria took place in 1953 (Pringle, 2005; Lowe-McConnell, 2006), but neither had been recorded in satellite Lake Malimbe as recently January 2003 (Katunzi & Kishe, 2004). Given the relatively recent timeline of the arrival of introduced species, future monitoring of genetic structure in these habitats may provide evidence of how shifts in density and time affect the frequency of hybridization.

### Morphological evaluation of hybrids

In natural systems, it can be possible to identify hybrid individuals on the basis of morphological characters. For example, F1 hybrids between the European cyprinids roach *Rutilus rutilus* (L. 1758) and bream *Abramis brama* (L. 1758) in Ireland can be identified using geometric morphometric analysis of body shape (Hayden et al., 2010), while hybrids of Atlantic salmon *Salmo salar* (L. 1758) and brown trout *Salmo trutta* (L. 1758) also typically exhibit intermediate morphology when measured using geometric morphometric approaches (Solem et al., 2014). We found that it was possible to separate purebred individuals of different species in sympatry using shape information, but hybrids overlapped in morphospace with parental individuals. Thus, we suggest that conclusive assignment of some *Oreochromis* hybrids may not be possible from geometric morphometric data of the gross body shape alone, although perhaps the method may be useful when used in combination with other phenotypic traits such melanin patterning.

### Biodiversity and fisheries implications

Hybridization can result in biodiversity loss through genetic swamping, where hybridization leads to the loss of unique genetic diversity, or demographic swamping, where the numeric increases in hybrids result in negative demographic consequences for the parental species (Todesco et al., 2016). The absence or low frequency of hybrids between introduced and indigenous species suggests neither of these scenarios are likely under current environmental regimes at our study sites. However, hybridization at low frequencies can lead to the introduction of novel alleles that either reduce fitness of native species (Muhlfield et al., 2009), or promote traits such as fast maturation and small body sizes that could compromise fish production. By contrast, hybridization can promote the sharing of beneficial alleles, reducing vulnerability to inbreeding and disease and facilitating increased niche width by both native and introduced populations (Hall, 2016; Pfennig et al., 2016). In principle, this can lead to net benefits to capture fisheries through improving survivorship. In *Oreochromis,* it is unclear if the presence of hybridization between native and non-native species has affected production in either capture fisheries or aquaculture, but these questions could be investigated through further work on the genomic composition of these *Oreochromis* communities, together with common-garden experiments investigating traits related to fish production.

### Concluding remarks

Fisheries in East Africa are essential for local livelihoods and food security (Muir et al., 2005; Heck et al., 2007; Musaka & Musonda, 2013), and the introduced species *O. niloticus* and *O. leucostictus* are now important components of demersal fisheries in northern Tanzania (e.g. Kolding et al., 2014). However, any benefits from further spread of introduced species in East Africa must be weighed against potential risks to biodiversity, existing stocks, and future potential fisheries yields. We propose that fisheries managers adopt the precautionary principle, that suggests future aquaculture and capture fisheries development should be based primarily on indigenous large-bodied species, unless there is compelling evidence that the economic and societal benefits will outweigh risks to biodiversity and existing artisanal fisheries. Further information on the likelihood of hybridization among species under different environmental conditions would help to guide policy and fisheries development in the region.

## Electronic supplementary material

Below is the link to the electronic supplementary material.

Supplementary material 1 (DOCX 41 kb)

## References

[CR1] Adams, D. C., M. L. Collyer, A. Kaliontzopoulou, & E. Sherratt. 2017. Geomorph: Software for geometric morphometric analyses. R package version 3.0.5. https://cran.r-project.org/package=geomorph.

[CR2] Agresti JJ, Seki S, Cnaani A, Poompuang S, Hallerman EM, Umiel N, Hulata G, Gall GAE, May B (2000). Breeding new strains of tilapia: development of an artificial center of origin and linkage map based on AFLP and microsatellite loci. Aquaculture.

[CR3] Angienda PO, Lee HJ, Elmer KR, Abila R, Waindi EN, Meyer A (2011). Genetic structure and gene flow in an endangered native tilapia fish (*Oreochromis esculentus*) compared to invasive Nile tilapia (*Oreochromis niloticus*) in Yala Swamp, East Africa. Conservation Genetics.

[CR4] Bailey RG, Churchfield S, Petr T, Pimm R (1978). The ecology of the fishes in Nyumba ya Mungu reservoir, Tanzania. Biological Journal of the Linnean Society.

[CR5] Bakhoum SA, Sayed-Ahmed MA, Ragheb EA (2009). Genetic evidence for natural hybridization between Nile tilapia (*Oreochromis niloticus*: Linnaeus 1757) and Blue tilapia (*Oreochromis aureus*: Steindachner 1864), in Lake Edku, Egypt. Global Veterinaria.

[CR6] Bartley DN, Rana K, Immink AJ (2001). The use of inter-specific hybrids in aquaculture and fisheries. Reviews in Fish Biology and Fisheries.

[CR7] Bolnick DI, Near TJ (2005). Tempo of hybrid inviability in centrarchid fishes (Teleostei: Centrarchidae). Evolution.

[CR8] Canonico CG, Arthington A, McCrary JK, Thieme ML (2005). The effect of introduced tilapias on native biodiversity. Aquatic Conservation: Marine and Freshwater Ecosystems.

[CR9] Clavero PM, Garcia-Bethou E (2005). Invasive species are a leading cause of animal extinctions. Trends in Ecology and Evolution.

[CR10] Cox JG, Lima SI (2006). Naïveté and an aquatic-terrestrial dichotomy in the effects of introduced predators. Trends in Ecology and Evolution.

[CR11] Crooks JA, Soulé ME, Sandlund OT, Schei PJ, Viken A (1999). Lag times in population explosions of invasive species: causes and implications. Invasive Species and Biodiversity Management.

[CR12] D’Amato ME, Esterhuyse MM, van der Waal BCW, Brink D, Volckaert FAM (2007). Hybridization and phylogeography of the Mozambique tilapia *Oreochromis mossambicus* in southern Africa evidenced by mitochondrial and microsatellite DNA genotyping. Conservation Genetics.

[CR13] Deines AM, Bbole I, Katongo C, Feder JL, Lodge DM (2014). Hybridisation between non-indigenous *Oreochromis niloticus* in the Kafue river, Zambia. African Journal of Aquatic Science.

[CR14] Deines AM, Wittmann ME, Deines JM, Lodge DM (2016). Tradeoffs among ecosystem services associated with global tilapia introductions. Reviews in Fisheries Science and Aquaculture.

[CR15] Doupé RG, Schaffer J, Knott MJ, Burrows DW (2009). How might an exotic fish disrupt spawning success in a sympatric native species?. Marine and Freshwater Research.

[CR16] Drake JM, Lodge DM (2003). Global hotspots of biological invasion: evaluating options for ballast-water management. Proceedings of the Royal Society London, Biology.

[CR17] Excoffier L, Lische HEL (2010). Arlequin suite ver 3.5: a new series of programs to perform population genetics analyses under Linux and Windows. Molecular Ecology Resources.

[CR18] FAO, 2012. The state of world fisheries and aquaculture 2012. Rome 200. http://www.fao.org/docrep/016/i2727e/i2727e00.htm

[CR20] Genner MJ, Nichols P, Carvalho GR, Robinson RL, Shaw PW, Turner GF (2007). Reproductive isolation among deep-water cichlid fishes of Lake Malawi differing in monochromatic male breeding dress. Molecular Ecology.

[CR19] Genner MJ, Connell E, Shechonge A, Smith A, Swanstrom J, Mzighani S, Mwijage A, Ngatunga BP, Turner GF (2013). Nile tilapia invades the Lake Malawi catchment. African Journal of Aquatic Science.

[CR21] Gregg RE, Howard JH, Shonhiwa F (1998). Introgressive hybridization of tilapia in Zimbabwe. Journal of Fish Biology.

[CR22] Hall RJ (2016). Hybridization helps colonizers become conquerors. Proceedings of the National Academy of Sciences USA.

[CR23] Hasselman DJ, Argo EE, McBride MC, Bentzen P, Schultz TF, Perez-Umphrey AA, Palkovacs EP (2014). Human disturbance causes the formation of a hybrid swarm between two naturally sympatric fish species. Molecular Ecology.

[CR24] Hayden B, Pulcini D, Kelly-Quinn M, O’Grady M, Caffrey J, McGrath A, Mariani S (2010). Hybridisation between two cyprinid fishes in a novel habitat: genetics, morphology and life-history traits. BMC Evolutionary Biology.

[CR25] Heck S, Béné C, Reyes-Gaskin R (2007). Investing in African fisheries: building links to the millennium development goals. Fish and Fisheries.

[CR27] Hill JE, Cichra CE (2005). Eradication of reproducing populations of convict cichlids, *Cichlasoma nigrofasciatum* (Cichlidae), in North-Central Florida. Florida Scientist.

[CR26] Hill JE, Sowards J (2015). Successful eradication of the non-native loricaridd catfish *Pterygolichthys disjunctivus* from the Rainbow River, Florida. Management of Biological Invasions.

[CR28] Hulme PE, Bacher S, Kenis M, Klotz S, Kühn I, Minchin D, Nentwig W, Olenin S, Panov V, Pergl J, Pyšek P (2008). Grasping at the routes of biological invasions: a framework for integrating pathways into policy. Journal of Applied Ecology.

[CR29] Jombart T, Ahmed I (2011). adegenet 1.3-1: new tools for the analysis of genome-wide SNP data. Bioinformatics.

[CR30] Kamal AHMM, Mair GC (2005). Salinity tolerance in superior genotypes of tilapia, *Oreochromis niloticus, Oreochromis mossambicus* and their hybrids. Aquaculture.

[CR31] Katunzi EFB, Kishe MA (2004). Changes in population structures of the major species in selected satellite lakes around Lake Victoria following changes in fishing effort. Tanzania Journal of Science.

[CR32] Klingenberg CP (2011). MorphoJ: an integrated software package for geometric morphometrics. Molecular Ecology Resources.

[CR33] Kolding, J., M. Medard, O. Mkumbo & P. A. M. van Zwieten, 2014. Status, trends and management of the Lake Victoria fisheries. In Inland fisheries evolution and management. Case studies from four continents. FAO: 49–62.

[CR34] Kopelman NM, Mayzel J, Jakobsson M, Rosenberg NA, Mayrose I (2015). Clumpak: a program for identifying clustering modes and packaging population structure inferences across K. Molecular Ecology Resources.

[CR35] Leprieur F, Beauchard O, Blanchet S, Oberdorff T, Brosse S (2008). Fish invasions in the world’s river systems, when natural process are blurred by human activities. PloS Biology.

[CR36] Leprieur F, Brosse S, Garcia-Berthou E, Oberdorff T, Olden JD, Townsend CR (2009). Scientific uncertainty and the assessment of risks posed by non-native freshwater fishes. Fish and Fisheries.

[CR37] Levin DA, Francisco-Ortega J, Jansen RK (1996). Hybridisation and the extinction of rare plant species. Conservation Biology.

[CR38] Levine JM, D’Antonio CM (2003). Forecasting biological invasions with increasing international trade. Conservation Biology.

[CR39] Lind CE, Brummett RE, Ponzoni RW (2012). Exploitation and conservation of fish genetic resources in Africa: issues and priorities for aquaculture development and research. Reviews in Aquaculture.

[CR40] Lowe S, Browne M, Boudjelas S, De Poorter M (2000). 100 of the world’s worst invasive alien species: a selection from the global invasive species database. Invasive Species Specialist Group, Aliens.

[CR41] Lowell SJ, Stone SF, Fernandez L (2006). The economic impact of aquatic invasive species: a review of the literature. Agricultural and Resource Economics Review.

[CR42] Lowe-McConnell R (2006). The Tilapia Trail.

[CR43] Maan ME, Seehausen O, Groothuis TG (2017). Differential survival between visual environments supports a role of divergent sensory drive in cichlid fish speciation. American Naturalist.

[CR44] McAndrew BJ, Roubal FR, Roberts RJ, Bullock AM, McEwen IM (1988). The genetics and histology of red, blond and associated colour variants in *Oreochromis niloticus*. Genetics.

[CR45] McGee MD, Reustle JW, Oufiero CE, Wainwright PC (2015). Intermediate kinematics produce inferior feeding performance in a classic case of natural hybridization. American Naturalist.

[CR46] Meyerson LA, Mooney HA (2007). Invasive alien species in an era of globalisation. Frontiers in Ecology and the Environment.

[CR47] Molnar JL, Gamboa RL, Revenga C, Spalding MD (2008). Assessing the global threat of invasive species to marine biodiversity. Frontiers in Ecology and the Environment.

[CR48] Moralee RD, van der Bank FH, van der Waal BCW (2000). Biochemical genetic markers to identify hybrids between the endemic *Oreochromis mossambicus* and the alien species, *O. niloticus* (Pisces: Cichlidae). Water SA.

[CR49] Muhlfeld CC, Kalinowski ST, McMahon TE, Taper ML, Painter S, Leary RF, Allendorf FW (2009). Hybridization rapidly reduces fitness of a native trout in the wild. Biology Letters.

[CR50] Muir, J. F., N. Gitongo, I. Omar, V. Poumogne & I. Radwan, 2005. Hidden harvests: unlocking the potential of aquaculture in Africa. Technical Review Paper. NEPAD-Fish for All Summit 22-25.

[CR51] Musaka CG, Musonda FF (2013). Contribution of small water bodies and small-holder aquaculture towards poverty allevation and enhancing household food security in Zambia. International Journal of Fisheries and Aquaculture.

[CR52] Ndiwa TC, Nyingi DW, Agnèse JF (2014). An important natural genetic resource of *Oreochromis niloticus* threatened by aquaculture activities in Loboi Drainage, Kenya. PloS ONE.

[CR53] Nilsson PA, Hulthén K, Chapman BB, Hansson LA, Brodersen J, Baktoft H, Vinterstare J, Brönmark C, Skov C (2017). Species integrity enhanced by a predation cost to hybrids in the wild. Biology Letters.

[CR54] Nyingi DW, Agnèse JF (2007). Recent introgressive hybridisation revealed by exclusive mtDNA transfer from *Oreochromis leucostictus* to *Oreochromis niloticus* in Lake Baringo, Kenya. Journal of Fish Biology.

[CR55] Perrings C, Dehnen-Schmutz K, Touza J, Williamson M (2005). How to manage biological invasions under globalisation. Trends in Ecology and Evolution.

[CR56] Pfennig KS, Kelly AL, Pierce AA (2016). Hybridization as a facilitator of species range expansion. Proceedings of the Royal Society London B.

[CR57] Pimentel D, Zuniga R, Morrison D (2005). Update on the environmental and economic costs associated with alien-invasive species in the United States. Ecological Economics.

[CR58] Pringle RM (2005). The Origins of the Nile Perch in Lake Victoria. BioScience.

[CR59] Pritchard JK, Stephens M, Donnelly P (2000). Inference of population structure using multilocus genotype data. Genetics.

[CR60] Rhymer JM, Simberloff D (1996). Extinction by hybridisation and introgression. Annual Review of Ecology and Systematics.

[CR61] Rognon X, Guyomard R (2003). Large extent of mitochondrial DNA transfer from *Oreochromis aureus* to *O. niloticus* in West Africa. Molecular Ecology.

[CR62] Rohlf, F. J., 2005. tpsDig, digitize landmarks and outlines, version 2.05. Department of Ecology and Evolution, State University of New York at Stony Brook.

[CR63] Romana-Eugia MRR, Ikeda M, Basiao ZU, Taniguchi N (2004). Genetic diversity in farmed Asian Nile and red hybrid tilapia stocks evaluated from microsatellite and mitochondrial DNA analysis. Aquaculture.

[CR64] Rosenfield JA, Nolasco S, Lindauer S, Sandoval C, Kodric-Brown A (2004). The role of hybrid vigor in the replacement of Pecos pupfish by its hybrids with sheepshead minnow. Conservation Biology.

[CR65] Sala OE, Chapin FS, Armesto JJ, Berlow E, Bloomfield J, Dirzo R, Huber-Sanwald E, Huenneke LF, Jackson RB, Kinzig A, Leemans R (2000). Global biodiversity scenarios for the year 2100. Science.

[CR66] Scribner KT, Page KS, Barton ML (2001). Hybridisation in freshwater fishes: a review of case studies and cytonuclear methods of biological inference. Reviews in Fish Biology and Fisheries.

[CR68] Seehausen O, van Alphen JJ, Witte F (1997). Cichlid fish diversity threatened by eutrophication that curbs sexual selection. Science.

[CR67] Seehausen O, Terai Y, Magalhaes IS, Carlton KL, Mrosso HD, Miyagi R, van der Slujis I, Schneider MV, Maan ME, Tachnida H, Imai H (2008). Speciation through sensory drive in cichlid fish. Nature.

[CR69] Solem Ø, Berg OK, Verspoor E, Hindar K, Karlsson SO, Koksvik J, Rønning L, Kjærstad G, Arnekleiv JV (2014). Morphological and genetic comparison between naturally produced smolts of Atlantic salmon, brown trout and their hybrids. Fisheries Management and Ecology.

[CR70] Stelkens RB, Schmid C, Selz O, Seehausen O (2009). Phenotypic novelty in experimental hybrids is predicted by the genetic distance between species in cichlid fish. BMC Evolutionary Biology.

[CR71] Taylor BW, Irwin RE (2004). Linking economic activities to the distribution of exotic plants. Proceedings of the National Academy of Sciences of the United States of America.

[CR72] Todesco M, Pascual MA, Owens GL, Ostevik KL, Moyers BT, Hübner S, Heredia SM, Hahn MA, Caseys C, Bock DG, Rieseberg LH (2016). Hybridization and extinction. Evolutionary Applications.

[CR73] Trewavas E (1983). Tilapiine fishes of the genera Sarotherodon, Oreochromis and Danakilia.

[CR74] Velema GJ, Rosenfeld JS, Taylor EB (2012). Effects of invasive American signal crayfish (*Pacifastacus leniusculus*) on the reproductive behaviour of threespine stickleback (*Gasterosteus aculeatus*) sympatric species pairs. Canadian Journal of Zoology.

[CR75] Westphal MI, Browne M, MacKinnon K, Noble I (2008). The link between international trade and the global distribution of invasive alien species. Biological Invasions.

[CR76] Williams SL, Grosholz ED (2008). The invasive species challenge in estuarine and coastal environments: marrying management and science. Estuaries and Coasts.

[CR77] Zengeya TA, Robertson MP, Booth AJ, Chimimba CT (2013). A qualitative ecological risk assessment of the invasive Nile tilapia, *Oreochromis niloticus* in a sub-tropical African river system (Limpopo River, South Africa). Aquatic Conservation: Marine and Freshwater Ecosystems.

