# Supplementary material for: Limited hybridization between introduced and Critically Endangered indigenous tilapia fishes in northern Tanzania
Source: Hydrobiologia. 2018 Apr 18;832(1):257–68. doi: 10.1007/s10750-018-3572-5 (PMC6394572; doi:10.1007/s10750-018-3572-5)
Supplement: Supplementary file 1 — Supplementary material 1 (DOCX 41 kb) [file 10750_2018_3572_MOESM1_ESM.docx]

**Supporting Information Table 1.** Microsatellite loci used in this study.

| Marker name | Genbank Accesson | Primer sequence (forward) | Primer sequence (reverse) | Motif | Dye | Source |
| --- | --- | --- | --- | --- | --- | --- |
|  |  |  |  |  |  |  |
| **OMO 043** | JX204857 | GGGGTCATTCGGTTTATTGGTTAT | AGGGCAGGTCACGGGTTCG | (TTTG)8 | PET | Liu *et al.,* 2013 |
| **OMO 093** | JX204891 | AAGCCCCACATAGACGACCAGAGA | CAGAAACGGTGCCTGTTCCAGAA | (CAT)8 | PET | Liu *et al.,* 2013 |
| **OMO 100** | JX204895 | CCTTCCCCACCACTACCCTCATAA | CCCGCCCACACCTGACGA | (ATT)18 | PET | Liu *et al.,* 2013 |
| **OMO 114** | JX204905 | ACGCCTTAATGCTGCCTTCAAGA | TGATGCTCACCCCGTTCCTCA | (GTT)11 | NED | Liu *et al.,* 2013 |
| **OMO 129** | JX204914 | TTGGCAGGCTAAGTACTATTTCAT | GAGCGAATGGTTGTCTGTCTCT | (CCAT)9 | 6-FAM | Liu *et al.,* 2013 |
| **OMO 161** | JX204924 | ACTTTGACAAAAGAAGTGTAACAA | AGGGGAGGAGAAAATAAACTGTAT | (TAA)10 | PET | Liu *et al.,* 2013 |
| **OMO 219** | JX204964 | ATCCCCTTCTTTCCATCCCTGTC | AAGGCCTCTGTGAGCTGATTGATT | (TTTTG)10 | 6-FAM | Liu *et al.,* 2013 |
| **OMO 229** | JX204973 | GCGACTTTTTCTTTGCACATTTTT | AACTGAACCGCCATCATAATCATC | (GTT)9 | 6-FAM | Liu *et al.,* 2013 |
| **OMO 248** | JX204987 | AAAGACACAAAGAGAAACTAATCA | GGATGAATATTTAAAATCAGTCAG | (TCA)9 | PET | Liu *et al.,* 2013 |
| **OMO 337** | JX205052 | TAGGAGAGGCATAGGTTGTCAAAT | CAAGAGTCTAGGAGGGAATCAAAA | (GTTT)7 | VIC | Liu *et al.,* 2013 |
| **OMO 391** | GR699257 | AGACATCTGTACGCTCTTTACGAA | AGTGCTAGAGGGAAGGGGCTGTA | (GAT)9 | VIC | Liu *et al.,* 2013 |
| **OMO 392** | GR698887 | CTGGCTTAACTTCTCTACTGGACA | TCTACTCAAAACTGGCAACAAAAC | (GAATA)7 | VIC | Liu *et al.,* 2013 |
| **OMO 397** | GR693794 | ACGCGTGTTTGAGATATTTAGATT | GAACAAACAAGGGGAGTGG | (GATT)7 | PET | Liu *et al.,* 2013 |
| **OM-01** | GU391020 | TTTAAAGTTACACAGCAGTACAAAG | TTGTAGCATTTCAACACAGTCTC | (GT)20 | 6-FAM | Saju *et al.,* 2010 |
| **OM-03** | GU391022 | CTTTTTAATGAGCAACTTTTAAGTC | TGTGAATTTGACAACTTCCTTTC | (GATA)47 | NED | Saju *et al.,* 2010 |
| **OM-04** | GU391022 | AGCTCAAAACCTCATACAAAGG | GCAGAGATGTCAGATGTTGTTC | (GACA)6 (GATA)16 | 6-FAM | Saju *et al.,* 2010 |
| **OM-09** | GU391028 | GGCTACAACACCTGGATGG | TTGGGCTTACTGAAGCTGAC | (GT)26 | VIC | Saju *et al.,* 2010 |
|  |  |  |  |  |  |  |

**Supporting Information Table 2.** Genetic variation with focal samples

| **Sample** | **Variable / Locus** | **OMO093** | **OMO114** | **OMO161** | **OMO219** | **OMO337** | **OMO391** | **OMO392** | **OMO397** | **OMO229** | **OMO129** | **OM-03** | **OM-01** | **OM-04** | **OM-09** | **OMO100** | **OMO043** | **OMO248** |
| --- | --- | --- | --- | --- | --- | --- | --- | --- | --- | --- | --- | --- | --- | --- | --- | --- | --- | --- |
|  |  |  |  |  |  |  |  |  |  |  |  |  |  |  |  |  |  |  |
| **Lake Malimbe** | N individuals | - | 52 | - | 55 | - | 56 | 52 | 55 | 53 | 51 | 41 | 54 | 48 | 23 | - | - | 56 |
|  | N alleles | - | 7 | - | 6 | - | 5 | 7 | 9 | 9 | 6 | 11 | 15 | 13 | 9 | - | - | 6 |
|  | H obs | - | 0.615 | - | 0.200 | - | 0.393 | 0.596 | 0.582 | 0.340 | 0.510 | 0.732 | 0.481 | 0.542 | 0.304 | - | - | 0.393 |
|  | H exp | - | 0.691 | - | 0.644 | - | 0.676 | 0.699 | 0.673 | 0.573 | 0.655 | 0.807 | 0.847 | 0.636 | 0.830 | - | - | 0.813 |
|  | P-value (HWE) | - | 0.002 | - | < 0.001 | - | < 0.001 | < 0.001 | < 0.001 | < 0.001 | 0.002 | 0.001 | < 0.001 | < 0.001 | < 0.001 | - | - | < 0.001 |
|  |  |  |  |  |  |  |  |  |  |  |  |  |  |  |  |  |  |  |
| **Kerenge** | N individuals | 40 | 40 | 40 | 40 | 40 | 40 | 37 | 40 | 40 | 40 | 40 | 38 | 38 | 40 | 31 | 40 | 40 |
|  | N alleles | 5 | 6 | 4 | 5 | 4 | 8 | 4 | 8 | 8 | 7 | 11 | 13 | 8 | 9 | 7 | 9 | 5 |
|  | H obs | 0.600 | 0.675 | 0.550 | 0.525 | 0.225 | 0.650 | 0.405 | 0.475 | 0.550 | 0.600 | 0.675 | 0.500 | 0.368 | 0.600 | 0.742 | 0.400 | 0.650 |
|  | H exp | 0.744 | 0.770 | 0.746 | 0.756 | 0.543 | 0.824 | 0.571 | 0.830 | 0.643 | 0.745 | 0.817 | 0.881 | 0.523 | 0.500 | 0.771 | 0.530 | 0.707 |
|  | P-value (HWE) | 0.010 | 0.020 | < 0.001 | < 0.001 | < 0.001 | < 0.001 | 0.009 | < 0.001 | 0.633 | 0.001 | 0.060 | < 0.001 | 0.042 | 0.993 | 0.039 | 0.009 | 0.009 |
|  |  |  |  |  |  |  |  |  |  |  |  |  |  |  |  |  |  |  |
| **Lake Kumba** | N individuals | 80 | 84 | 81 | 75 | 84 | 84 | 81 | 84 | 84 | 71 | 74 | 73 | 75 | 74 | 74 | 74 | 74 |
|  | N alleles | 5 | 5 | 3 | 5 | 4 | 6 | 4 | 6 | 7 | 3 | 8 | 7 | 6 | 6 | 6 | 4 | 5 |
|  | H obs | 0.525 | 0.571 | 0.457 | 0.453 | 0.298 | 0.500 | 0.444 | 0.476 | 0.333 | 0.296 | 0.676 | 0.493 | 0.107 | 0.878 | 0.297 | 0.081 | 0.446 |
|  | H exp | 0.708 | 0.664 | 0.625 | 0.628 | 0.627 | 0.699 | 0.642 | 0.706 | 0.508 | 0.363 | 0.767 | 0.657 | 0.298 | 0.599 | 0.403 | 0.129 | 0.545 |
|  | P-value (HWE) | < 0.001 | < 0.001 | < 0.001 | < 0.001 | < 0.001 | < 0.001 | < 0.001 | < 0.001 | < 0.001 | 0.297 | < 0.001 | < 0.001 | < 0.001 | < 0.001 | < 0.001 | 0.001 | < 0.001 |
|  |  |  |  |  |  |  |  |  |  |  |  |  |  |  |  |  |  |  |
| **Nyumba-ya-Mungu** | N individuals | 35 | 37 | 34 | 34 | 36 | 36 | 33 | 37 | 36 | 22 | 33 | 29 | 35 | 34 | 35 | 36 | 35 |
|  | N alleles | 4 | 7 | 5 | 6 | 4 | 8 | 6 | 9 | 11 | 9 | 12 | 11 | 14 | 8 | 11 | 9 | 6 |
|  | H obs | 0.143 | 0.405 | 0.471 | 0.176 | 0.056 | 0.361 | 0.273 | 0.595 | 0.722 | 0.591 | 0.818 | 0.207 | 0.686 | 0.882 | 0.600 | 0.222 | 0.371 |
|  | H exp | 0.603 | 0.677 | 0.708 | 0.512 | 0.560 | 0.766 | 0.680 | 0.793 | 0.853 | 0.852 | 0.887 | 0.644 | 0.894 | 0.663 | 0.759 | 0.490 | 0.700 |
|  | P-value (HWE) | < 0.001 | < 0.001 | < 0.001 | < 0.001 | < 0.001 | < 0.001 | < 0.001 | < 0.001 | < 0.001 | < 0.001 | 0.005 | < 0.001 | < 0.001 | 0.469 | 0.060 | < 0.001 | < 0.001 |
|  |  |  |  |  |  |  |  |  |  |  |  |  |  |  |  |  |  |  |
| **Pangani Falls Dam** | N individuals | 41 | 42 | 40 | 41 | 42 | 42 | 42 | 42 | 41 | 35 | 35 | 41 | 36 | 41 | 42 | 41 | 42 |
|  | N alleles | 7 | 7 | 5 | 4 | 4 | 7 | 5 | 9 | 11 | 7 | 13 | 15 | 12 | 12 | 8 | 10 | 6 |
|  | H obs | 0.439 | 0.571 | 0.425 | 0.439 | 0.214 | 0.619 | 0.548 | 0.619 | 0.805 | 0.514 | 0.514 | 0.537 | 0.556 | 0.707 | 0.571 | 0.439 | 0.524 |
|  | H exp | 0.784 | 0.830 | 0.787 | 0.751 | 0.696 | 0.824 | 0.711 | 0.854 | 0.875 | 0.817 | 0.899 | 0.925 | 0.775 | 0.572 | 0.806 | 0.669 | 0.730 |
|  | P-value (HWE) | < 0.001 | < 0.001 | < 0.001 | < 0.001 | < 0.001 | 0.001 | < 0.001 | 0.001 | < 0.001 | < 0.001 | < 0.001 | < 0.001 | < 0.001 | 0.999 | < 0.001 | < 0.001 | 0.032 |
|  |  |  |  |  |  |  |  |  |  |  |  |  |  |  |  |  |  |  |

**Supporting Information Table 3.** Genetic variation with reference samples

| **Sample** | **Variable / Locus** | **OMO093** | **OMO114** | **OMO161** | **OMO219** | **OMO337** | **OMO391** | **OMO392** | **OMO397** | **OMO229** | **OMO129** | **OM-03** | **OM-01** | **OM-04** | **OM-09** | **OMO100** | **OMO043** | **OMO248** |
| --- | --- | --- | --- | --- | --- | --- | --- | --- | --- | --- | --- | --- | --- | --- | --- | --- | --- | --- |
|  |  |  |  |  |  |  |  |  |  |  |  |  |  |  |  |  |  |  |
| ***O. leucostictus* (Ref)** | N individuals | - | 5 | - | 5 | - | 5 | 2 | 5 | 5 | 5 | 3 | 4 | - | - | - | 4 | 4 |
|  | N alleles | - | 1 | - | 2 | - | 2 | 1 | 2 | 2 | 2 | 3 | 4 | - | - | - | 2 | 3 |
|  | H obs | - | 0.000 | - | 0.200 | - | 0.200 | 0.000 | 0.200 | 0.600 | 0.400 | 0.667 | 0.500 | - | - | - | 0.000 | 0.500 |
|  | H exp | - | - | - | 0.200 | - | 0.467 | - | 0.200 | 0.467 | 0.533 | 0.733 | 0.821 | - | - | - | 0.571 | 0.714 |
|  | P-value (HWE) | - | - | - | 1.000 | - | 0.334 | - | 1.000 | 1.000 | 1.000 | 1.000 | 0.314 | - | - | - | 0.086 | 0.316 |
|  |  |  |  |  |  |  |  |  |  |  |  |  |  |  |  |  |  |  |
| ***O. esculentus* (Ref)** | N individuals | 7 | 7 | 7 | 7 | 7 | 7 | 7 | 7 | 7 | 7 | 2 | 6 | 6 | 6 | - | 7 | 7 |
|  | N alleles | 2 | 2 | 2 | 2 | 1 | 2 | 5 | 2 | 5 | 2 | 1 | 2 | 1 | 1 | - | 2 | 1 |
|  | H obs | 0.143 | 0.143 | 0.143 | 0.000 | 0.000 | 0.000 | 1.000 | 0.143 | 0.571 | 0.714 | 0.000 | 0.167 | - | - | - | 0.000 | 0.000 |
|  | H exp | 0.143 | 0.363 | 0.143 | 0.264 | - | 0.264 | 0.703 | 0.143 | 0.505 | 0.495 | - | 0.167 | - | - | - | 0.264 | - |
|  | P-value (HWE) | 1.000 | 0.230 | 1.000 | 0.076 | - | 0.076 | 0.334 | 1.000 | 1.000 | 0.440 | - | 1.000 | - | - | - | 0.077 | - |
|  |  |  |  |  |  |  |  |  |  |  |  |  |  |  |  |  |  |  |
| ***O. niloticus* (Ref)** | N individuals | 7 | 8 | 8 | 8 | 8 | 8 | 8 | 8 | 8 | 8 | 8 | 8 | - | 8 | 8 | 8 | 8 |
|  | N alleles | 2 | 4 | 2 | 3 | 1 | 4 | 2 | 3 | 4 | 3 | 5 | 7 | - | 7 | 4 | 3 | 4 |
|  | H obs | 0.571 | 0.625 | 0.375 | 0.875 | 0.000 | 0.875 | 0.250 | 0.250 | 0.500 | 0.625 | 0.500 | 0.625 | - | 0.625 | 0.750 | 0.500 | 0.500 |
|  | H exp | 0.440 | 0.675 | 0.458 | 0.708 | - | 0.750 | 0.400 | 0.242 | 0.692 | 0.492 | 0.708 | 0.792 | - | 0.775 | 0.575 | 0.425 | 0.642 |
|  | P-value (HWE) | 1.000 | 0.477 | 1.000 | 0.840 | - | 1.000 | 0.384 | 1.000 | 0.149 | 1.000 | **0.008** | 0.296 | - | 0.420 | 1.000 | 1.000 | 0.685 |
|  |  |  |  |  |  |  |  |  |  |  |  |  |  |  |  |  |  |  |
| ***O. jipe* (Ref)** | N individuals | 13 | 13 | 13 | 13 | 13 | 13 | 13 | 13 | 13 | 1 | 13 | 12 | 13 | 13 | 12 | 13 | 13 |
|  | N alleles | 1 | 3 | 1 | 1 | 2 | 4 | 5 | 2 | 7 | 2 | 2 | 4 | 8 | 2 | 3 | 4 | 2 |
|  | H obs | 0.000 | 0.615 | 0.000 | 0.000 | 0.231 | 0.231 | 0.538 | 0.000 | 0.769 | 1.000 | 0.154 | 0.417 | 0.769 | 0.462 | 0.667 | 0.385 | 0.538 |
|  | H exp | - | 0.465 | - | - | 0.508 | 0.551 | 0.591 | 0.148 | 0.729 | 1.000 | 0.443 | 0.612 | 0.809 | 0.369 | 0.583 | 0.452 | 0.508 |
|  | P-value (HWE) | - | 0.631 | - | - | 0.087 | **0.005** | 0.829 | **0.039** | 0.681 | 1.000 | **0.033** | 0.343 | 0.131 | 1.000 | 0.774 | 0.116 | 1.000 |
|  |  |  |  |  |  |  |  |  |  |  |  |  |  |  |  |  |  |  |
| ***O. korogwe* (Ref)** | N individuals | 39 | 40 | 39 | 33 | 40 | 40 | 40 | 40 | 40 | 39 | - | 35 | 35 | 35 | 36 | 35 | 35 |
|  | N alleles | 2 | 2 | 2 | 3 | 1 | 1 | 3 | 3 | 1 | 1 | - | 3 | 8 | 3 | 3 | 1 | 1 |
|  | H obs | 0.333 | 0.325 | 0.077 | 0.212 | 0.000 | 0.000 | 0.450 | 0.575 | 0.000 | 0.000 | - | 0.714 | 0.829 | 0.971 | 0.417 | 0.000 | 0.000 |
|  | H exp | 0.345 | 0.276 | 0.075 | 0.246 | - | - | 0.376 | 0.631 | - | - | - | 0.568 | 0.819 | 0.533 | 0.446 | - | - |
|  | P-value (HWE) | 1.000 | 0.564 | 1.000 | **0.028** | - | - | 0.598 | 0.443 | - | - | - | 0.213 | 0.990 | **< 0.001** | 0.560 | - | - |
|  |  |  |  |  |  |  |  |  |  |  |  |  |  |  |  |  |  |  |
